# Vitamin A deficiency: what eye health workers can do

**Published:** 2013

**Authors:** Babajide Adebisi, Leigh Evelyn Jaschke, Heather Ilene Katcher, Jessica Blankenship

**Affiliations:** Nutrition and Fortification Programme Manager, Helen Keller International (HKI) Nigeria; Regional Programme Coordinator: Vitamin A Supplementation Programme, HKI Africa Regional Office; Regional Monitoring and Evaluation Officer, HKI Africa Regional Office; Regional Micronutrient Advisor, HKI Africa Regional Office jblankenship@hki.org

**Figure:**
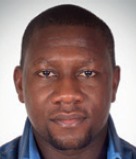
Babajide Adebisi

**Figure:**
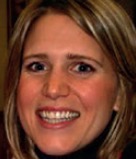
Leigh Evelyn Jaschke

**Figure:**
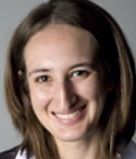
Heather Ilene Katcher

**Figure:**
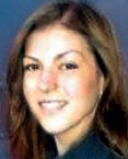
Jessica Blankenship

## Work with colleagues

Talk to the nurses and staff who work in maternal and child health clinics nearby. Ask what they know about vitamin A deficiency (VAD) and whether they are aware that it can lead to blindness. Encourage them to pass this information on to parents and to check that all children who are eligible receive their supplements at the correct time. Find out whether they are aware of – or involved in – any preventive vitamin A supplementation programmes.

Talk to colleagues in the paediatric ward of your hospital to make sure they have adequate supplies of high-dose vitamin A to treat children with severe diarrhoea, malnutrition and measles (as shown in Table 1, page 68), as these children are at high risk of VAD and blindness.

## Change your own practices

Consider making it routine practice to make a note in the medical records when each child last had a dose of vitamin A, and whether he or she has been immunised against measles. Do this for **all** children aged under 5 years who present to the clinic, regardless of the reason they present. If needed, talk to the child's parent or carer about the importance of immunisation and vitamin A supplementation.

If health talks are part of routine practice in the eye clinic or during outreach, include a ‘How to keep your child's eyes healthy’ session for parents and carers. This could include the importance of measles immunisation and vitamin A supplementation to prevent corneal scarring, as well as regular and routine face washing to prevent trachoma.

If you find a child with suspected VAD, talk to someone involved in a preventive supplementation programme – they may need to act on this information as there are likely to be other children in the community who are vitamin A deficient.

## Talk to families

First, find out about:

- which vitamin A-rich foods are sold in the local market at different times of the year
- local customs that might stop mothers giving their children vitamin A-rich foods
- local customs concerning the diet given to children when they are sick
- words in the local language for night blindness.

**Figure:**
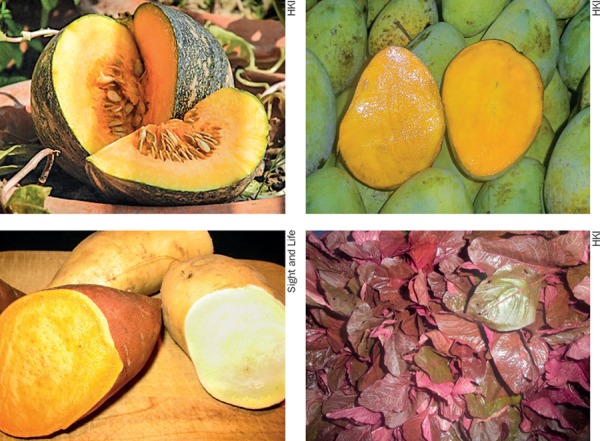
Figure 1. Examples of vitamin A-rich fruits and vegetables. Young children need 2–3 helpings of such foods per day.

Get as many of the following as possible to show to parents:

- vitamin A capsules
- vitamin A and/or multi-micronutrient powders (MNPs) (see page 69)
- locally available fortified foods
- photographs or real examples of foods rich in vitamin A (eggs, orange sweet potatoes, mangoes).

Discuss with families:

- the special needs of young children and pregnant and breastfeeding mothers for vitamin A-rich foods. Explain the risks of not eating enough vitamin A-rich foods
- which vitamin A-rich foods are available to them (this will depend on what can be grown or purchased in the area and the cost of these foods)
- which vitamin A-rich foods young children like and which are affordable
- any reasons why available vitamin A rich foods might not be eaten.

Ask the following questions:

- Have the parents ever noticed that their young child does not walk around or play after the sun has gone down, or when they are in a dark place? This may be ‘night blindness’ (use the local term if available) due to VAD (page 66). If the child has night blindness, ensure that he/she receives high-dose vitamin A supplementation (Table 1, page 68).
- Did the mother ever have night blindness during pregnancy or while she was breastfeeding? If so then she is likely to be deficient.

Encourage families to try available vitamin A-rich foods and to add a little oil to the food to aid absorption.

If vitamin A-rich foods are not available in the community, recommend that families grow foods such as dark green leafy vegetables or orange-fleshed sweet potatoes. If they do not own much land, point out that some vitamin A-rich foods, such as squashes, can be grown so they climb over the roofs of homes, using virtually no land.

Show families how to prepare foods in ways that children like. For example:

- chop and mash green leaves with oil-rich food (e.g. groundnuts) and staple starches
- mash carrots or orange-fleshed sweet potatoes.

## Advice for different ages

### Children younger than 6 months

Mothers should be encouraged to:

- start breastfeeding within 1 hour of giving birth. This ensures that their baby receives colostrum, which is an excellent source of vitamin A.
- Colostrum is full of nutrients, including vitamin A and other compounds, such as immunoglobulins, that help prevent infection
- exclusively breastfeed their baby until he/she is 6 months old and continue breastfeeding for at least 2 years, as breast milk is a very important source of vitamin A. Tell the mother: ‘There are important nutrients in breast milk including vitamin A, which help to keep your baby healthy and prevent measles and diarrhoea.’
- eat plenty of vitamin A rich foods themselves to ensure that their breast milk provides their infant with enough vitamin A as well as meeting their own health needs.

### Children aged 6 to 12 months

Mothers should be encouraged to continue to breastfeed and to feed their baby complementary foods that are rich in vitamin A. Even small children will eat these foods if they are given in small, frequent amounts, and are mashed up (perhaps mixed with expressed breast milk) to make them more appealing.

### Children aged around 9 months

Mothers should be encouraged to have their child immunised against measles. Measles is a very important cause of severe vitamin A deficiency which can lead to damage to their child's eyes. If their child is too sick, or they are unable to visit the clinic at 9 months, then the child can be taken to the clinic for immunisation as soon as he or she is well enough.

## Fortified foods

Encourage people to eat or cook with foods fortified with vitamin A if they are available, as this can help to prevent VAD.

Foods that are commonly fortified with vitamin A include vegetable oil, margarine, flour and sugar. Most countries that have adopted fortification have made it mandatory that staple foods are fortified.

In addition to staple foods fortified with vitamin A, home fortification using MNPs are an excellent way to increase the amount of vitamin A that young children eat. This approach is particularly useful if vitamin A-rich foods are not available in the community and complementary foods are poor in vitamin A (page 67).

**Figure:**
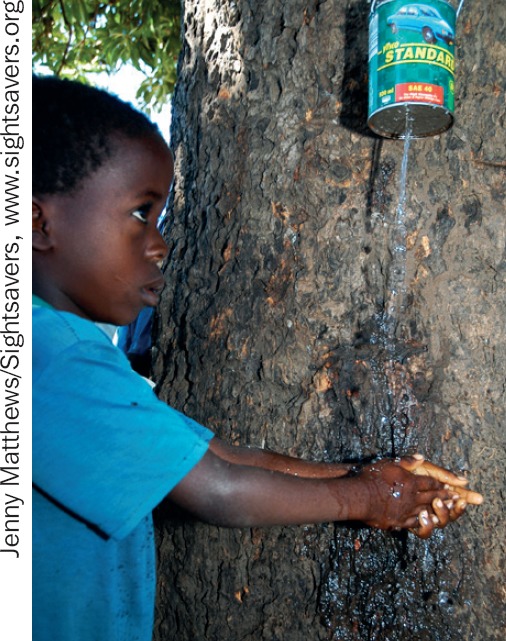
Hand Washing

## Hygiene

Help families to practice good hygiene and sanitation.

Because infections, especially diarrhoea, play an important role in undernutrition and VAD, health workers should ensure that families understand the importance of clean water and hygienic practices for good health.

Proper disposal of human and animal waste (including faeces) should be promoted as well as clean and safe preparation of foods. Mothers and carers should wash their hands with soap before preparing any food, and after defecating or cleaning a child after defecation. If water is scarce, show them how to make a very small hole in a tin and how to wash their hands and the hands of their children using very little water.

**Figure:**
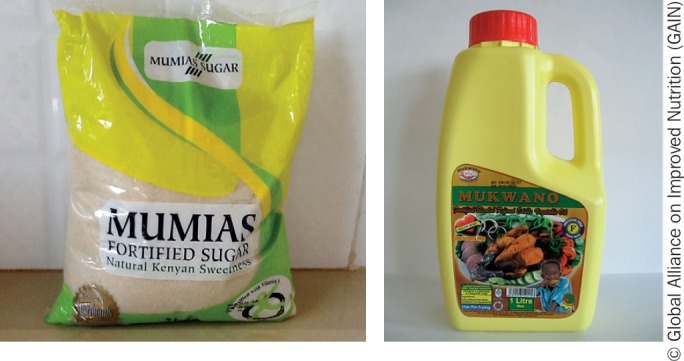
Show parents examples of locally available fortified foods. Pictured here are fortified sugar (left) and fortified cooking oil

Good hygiene practices will also reduce the transmission of intestinal worms, which contribute to undernutrition in general.

## Other ways to reduce vitamin A deficiency

If families have many children who are born close together, the mother's ability to breastfeed each one for long enough is reduced. Encourage families to use family planning methods to space their children at least 2 years apart.^1^

Sick children need even more nutritious foods than children who are well. Encourage mothers to feed children healthy foods – including those rich in vitamin A (see Figure 1 and the panel on page 65) – during periods of illness and/or diarrhoea.

**Shade drying**

Shade drying requires full air circulation. It should not be undertaken inside conventional buildings but in a purpose-built open-sided shed for shade drying. Most foods to be dried (e.g. mangoes, sweet potatoes and carrots) are sliced, as sliced food generally dries faster. The slices should be only about 1 cm thick so that they dry thoroughly and quickly. Leafy vegetables, such as amaranth, are dried whole because they are thin. The food should be placed on mats or trays, well off the ground in order to avoid contamination from dust or soil (see Figure 1). Turn over the slices daily to ensure that the food dries quickly. To store well, the slices should be quite dry. Fruits, however, need only be dried until they are leathery, as their higher sugar content acts as a preservative.

**Figure:**
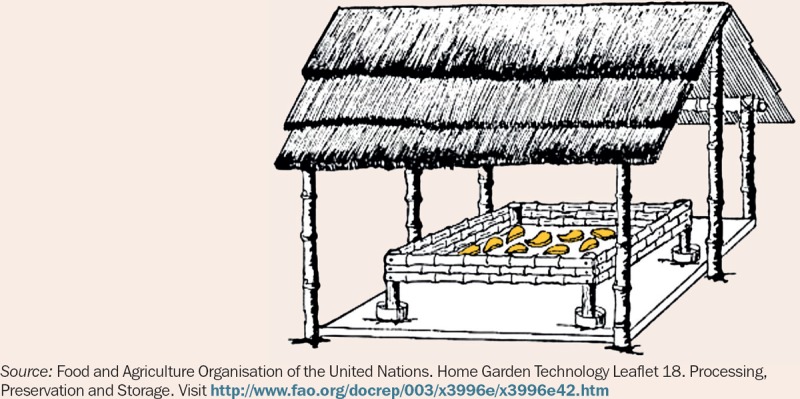
Figure 1. Placing the drying rack on a pedestal standing in a container of water prevents crawling insects from reaching the food
